# *In Vitro* Release of 5-Fluorouracil and Methotrexate from Different Thermosensitive Chitosan Hydrogel Systems

**DOI:** 10.1208/s12249-020-01672-6

**Published:** 2020-05-13

**Authors:** Ahmed M. Mohammed, Shaaban K. Osman, Khaled I. Saleh, Ahmed M. Samy

**Affiliations:** 1 Department of Pharmaceutics and Industrial pharmacy, College of Pharmacy, Al-Azhar University, Assiut, Egypt; 2 Department of Pharmaceutics and Industrial pharmacy, College of Pharmacy, Al-Azhar University, Cairo, Egypt

**Keywords:** thermosensitive, chitosan, *In vitro*, 5-fluorouracil, methotrexate and hydrogel

## Abstract

5-Fluorouracil is a member of cytotoxic drugs with poor selectivity to cancer cells. Currently, systemic administration of this anti-cancer drug (oral or injection) exposes normal tissues to the drug-induced toxicity. Nowadays, attention has been greatly directed towards *in situ* gel-forming systems that can be injected into the affected tissues in its sol form with a minimally invasive technique. More specifically, chitosan hydrogel systems were in focus due to their antibacterial effect as well as their biodegradable, biocompatible, and mucoadhesive properties. In the present work, 5-fluorouracil was loaded on various thermosensitive chitosan hydrogel systems cross linked with different linking agents like β-glycerophosphate, pluronic F127, and hydroxyapatite. Also, methotrexate was added to 5-fluorouracil in order to gain its previously reported synergistic effects. Firstly, a compatibility study was performed using UV-spectrophotometric, infrared spectroscopy (FTIR) and differential scanning calorimetry (DSC) techniques to exclude the possibility of any physical or chemical interactions between the selected drugs and excipients. The prepared hydrogel systems were characterized for their physicochemical properties including organoleptic, pH, syringeability and injectability, viscosity, and gelation temperature (*T*_gel_) by various analysis techniques. Moreover*,* the *in vitro* release behavior of 5-fluorouracil and methotrexate was determined with a modified analytical method. The results indicated that chitosan hydrogel system cross-linked with a combination of β- glycerophosphate, and 10 % pluronicF127 (F4) showed the most suitable physicochemical properties and release profile. Accordingly, this formula can be considered as a missionary system for localized sustained delivery of cytotoxic drugs.

## INTRODUCTION

**Fig. 1:**
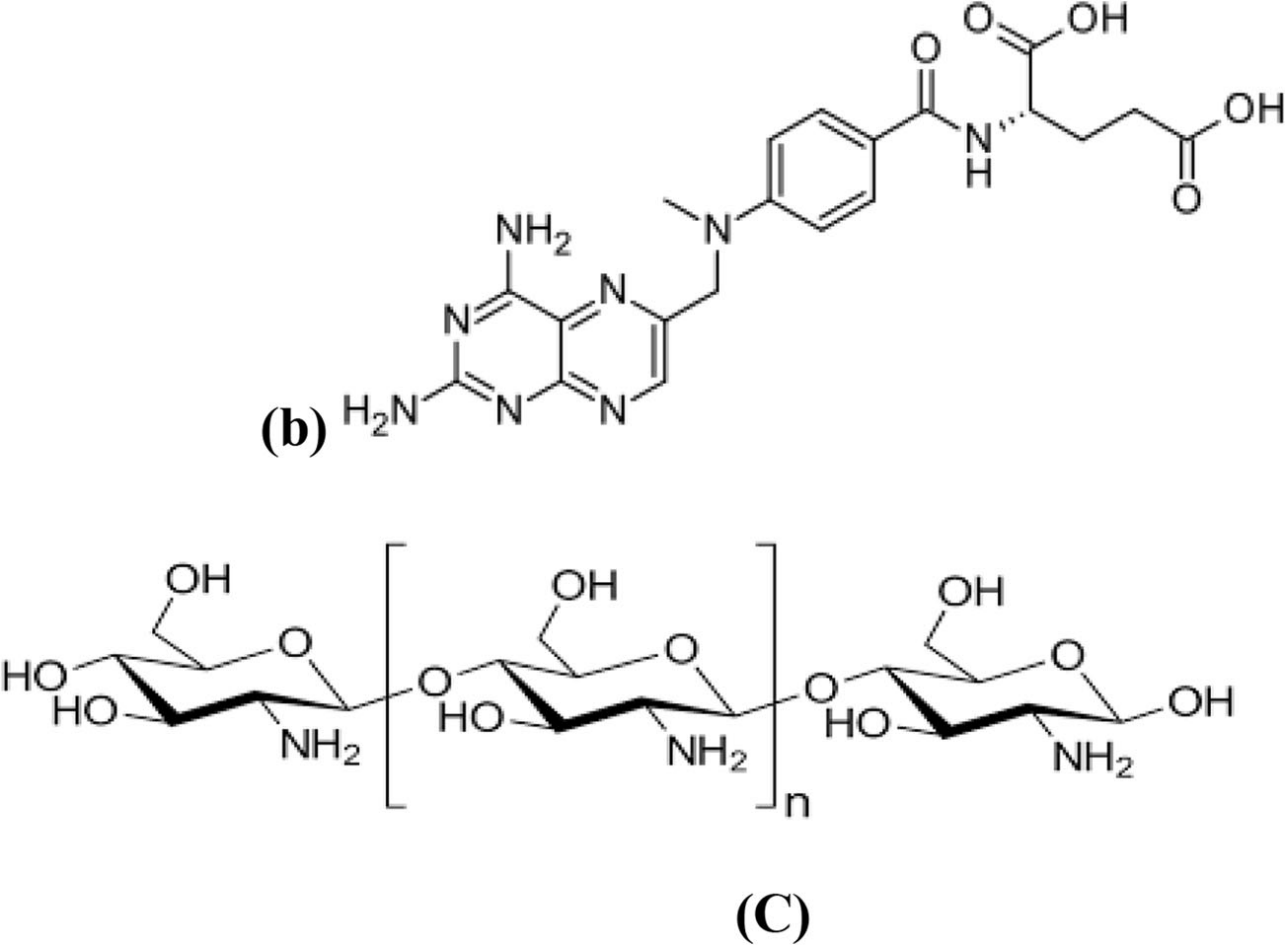
Chemical structure of 5-fluorouracil (**a**), methotrexate (**b**) and chitosan (**c**)

Current systemic administration of anti-cancer drugs exposes normal tissues to the drug-induced toxicity. This is because most cytotoxic drugs are non-selective and cannot distinguish between cancer and normal cells [1]. In addition, rapid release of the loaded dose causes rapid elevation of the drug level with unintended side effects at the peak followed by shape declining and insufficient therapeutic effect at the low drug level [2]. 5-fluorouracil (5-FU) is an anti-cancer drug having structural similarity to pyrimidine bases (Fig. 1), interfering with thymidylate synthase enzyme action and preventing DNA replication [3]. It was reported that pretreatment with methotrexate (MTX) increases 5-FU uptake by cells, due to inhibition of purine synthesis by MTX as a result of increased levels of intracellular phosphoribosyl-pyrophosphate. Inhibition of purine synthesis will result in accumulation of N5,N10-methylene tetrahydrofolate which is an essential factor for tight binding of 5-fluorouracil active metabolite to thymidylate synthetase enzyme (TS) [4, 5].

To overcome the problem of poor selectivity, systemic administration of these anti-cancer drugs has been replaced by localized targeting and controlled drug delivery systems and it shows promising results in the fields of chemotherapy [6].

Different sustained-release injectable systems were basically designed like implants [7, 8], nano and microparticles [7, 9], and liposomes [10] to provide localized drug delivery. However, these systems show limited loading capacity and require further surgical operations to insert it at the tumor site which results in extra risks and costs of these systems [11]. These problems have directed the researchers towards injectable biopolymer-based hydrogels for localized drug delivery.

Chitosan (CH), a natural biopolymer with similar structure and bioactivity to glycosaminoglycan, has been used as injectable hydrogel due to its antibacterial, mucoadhesive, biocompatible, and biodegradable properties [12]. Different chemically inert cross-linking agents like β-glycerophosphate, Pluronic F127, and hydroxyapatite (Ca_5_ (PO_4_)_3_ OH) were used in preparation of chitosan hydrogel in order to improve its physicochemical properties of chitosan hydrogel [13–15].

The present work was aiming to formulate a novel 5-FU with MTX systems in different biodegradable chitosan hydrogels for localized injection at the cancer tissue using different cross-linking agents like β-glycerophosphate (β-GP), pluronicF127 (Pl F127), and hydroxyapatite (HA). The compatibility study was achieved by using UV-spectrophotometric, infrared spectroscopy (FTIR), and differential scanning calorimetry (DSC) techniques for detection of any drug-excipient interactions. The formulated hydrogels were characterized by different analytical techniques for their physicochemical properties including drug content, pH, syringeability and injectability, viscosity, and gelation temperature (*T*_gel_). Moreover, an analytical procedure was developed for quantitative determination of 5-FU in the presence of MTX in the release medium. The most suitable formulae will be selected for further studies.

## MATERIALS AND METHODS

### Materials

5-fluorouracil (5-FU) was purchased from Applichem for pharmaceuticals Co., Gmbh (Germany). Medium-molecular-weight Chitosan of 190 KDa, pluronic F127, and β-glycerophosphate (β-GP) were purchased from Fluka BioChemika (Switzerland). Methotrexate (MTX) was obtained in lyophilized form from Haupt Pharma GmbH-Chemajet Co., (Germany). Acetic acid, calcium chloride, and potassium dihydrogen phosphate were obtained from Al-Nasr Pharm. Chem. Co., (Cairo, Egypt). All other solvents and materials were of high analytical grade.

### Compatibility Studies

Compatibility studies for drugs and excipients were performed to exclude the possibility of chemical interactions which may affect the therapeutic activity of the drugs [16]. Compatibility study was carried out by using UV-spectrophotometric, infrared spectroscopy (FTIR), and differential scanning calorimetry (DSC).

#### Scanning Ultraviolet Absorption Study

A 1% solution of each excipient in distilled water was scanned in the presence and absence of the drugs using distilled water as a blank. This study was done to indicate the presence of any interference caused by the investigated excipients on the maximum absorbance of the drug in the used dilution range [17]. The maximum absorbance was measured spectrophotometrically using UV-visible spectrophotometer (UV-1601, Shimadzu, Japan).

#### FTIR Spectroscopy

The IR absorption spectra of pure components and physical mixture (1:1 ratio) were measured using IR-spectrophotometer (IR-476, Shimadzu, Japan) at a range of 4000–400 cm^−1^ [18]. The samples were compressed into KBr discs by the aid of IR compression machine [19].

#### Differential Scanning Calorimetry

DSC study was performed using DSC-Thermal analyzer (DSC-T50, Shimadzu, Japan). Samples were carefully weighed into aluminum pans and sealed. The temperature range for the thermogram was 30 to 350°C, and the samples were heated at rate of 10°C/min [20].

#### UV-Spectrophotometric Analysis of 5-FU in the Presence of MTX

There are some instances in which the presence of one species in a sample does not interfere with the measurement of another one in the same sample [21]. In the present work, the absorption of light by the components of the sample solution is additive. Then, by choosing wavelengths where the absorption of the first drug is strong and the second one is weak, it is still possible to determine their concentrations [21]. Firstly, the absorption spectrum of the two drugs individually was determined and chooses wavelengths which differentiate their response (λ_max_). Then, Beer’s Law curve over range of concentrations for each drug was constructed. The various absorptivity constants (*k*s) are determined from Beer’s Law plots for the separate components at the two wavelengths which are chosen for the analysis. With a two-unknown-component mixture, two mathematical equations were generated and solved simultaneously to obtain the concentrations of the two unknown components [17].

### Preparation of Thermosensitive Chitosan Hydrogel Systems

Chitosan solution was prepared according to Chenite et al.’s method [22]. Briefly, medium molecular weight chitosan of 190 KDa (1.8% w/w) was dissolved in 0.1 M acetic acid. The chitosan powder was sprinkled over the solvent under stirring for 30 min for complete mixing. Afterward, cold aqueous solutions of different cross-linking agents including β-GP (35% w/w), pluronic F127 (20% w/w), or hydroxyapatite (2.63% w/w) were prepared and chilled drop wise along with the previously prepared chitosan solutions (1:1 ratio) in an ice bath for 15 min, as presented in Table I. Finally, the obtained chitosan thermosensitive hydrogels (F1, F2, F3, and F4) were stored at 8°C [14, 15].

**Table I:** Percent Composition (w/w) of Chitosan Hydrogel Formulations Containing Different Cross-Linking Agents

Formulation code	Chitosan solution	Cross-linking agent solutions		
		β-Glycerophosphate	Pluronic F127	Hydroxyapatite
F1	1.8	35	–	–
F2	1.8	–	20	–
F3	1.8	–	–	2.63
F4	1.8	35	10	–

### Drug Loading

The method of drug loading greatly affects the release rate and bioavailability of the drugs. In the present work, both 5-FU and MTX were added to chitosan solution at concentration of 0.1 and 0.05% w/w, respectively, with agitating until it was dissolved thoroughly [23].

### Characterization of Chitosan Hydrogels

#### Drug Content

One-gram sample containing amount equivalent to 1 mg and 0.5 mg of 5-FU and MTX, respectively, was taken and dissolved in a sufficient quantity of distilled water. The obtained solution was filtered by using Whatman filter paper. The filtrate was appropriately diluted with distilled water, and the drug content was determined spectrophotometrically at two λ_max_ (260 and 302) using UV-Visible spectrophotometer [23].

#### Visual Inspection (Organoleptic Properties)

The prepared systems were visually examined in sol and gel forms for their purity, homogeneity, fluidity, and phase separation [24].

#### pH Determination

The pH of the prepared hydrogel systems was simply measured by immersing the probe of the pH meter into the samples using Ama Digital pH meter (Ama Co., Germany) [25].

#### Viscosity Measurement

At room temperature (25 ± 1°C), the viscosity of the prepared chitosan hydrogel solutions was measured using an Ostwald U tube capillary viscometer. Briefly, liquid is introduced into the viscometer until the level reaches the mark. The viscometer is fixed vertically in a thermostatic bath and allowed to attain the required temperature. The sample volume is adjusted and the liquid is sucked or blown into the other arm until the liquid is just above the mark. The suction or pressure is released, and the time taken for the bottom of the liquid to fall from the top mark to the bottom mark is noted [26].

At 37°C, the viscosity of chitosan hydrogels was measured with Brookfield DV-III ultra viscometer using T-bar spindle (T-D 94) at 50 rpm [27].

#### Syringeability and Injectability

Syringeability and injectability are of particular significance for any parenteral dosage forms. Suitable consistency is necessary in order to extrude the gel solution from the needle into the body in a proper manner. Practically, syringeability test was simply performed by filling syringe-needle system with the liquid preparations and injecting the solution into a piece of meat by the aid of finger pressure at room temperature [28].

#### Sol-Gel Transition Temperature Measurement

The cold solutions of different hydrogel systems were heated on water bath in range 8 to 40°C at constant rate with continuous stirring. The temperature at which the magnetic bar stopped to move due to gelation was recorded as the gelation temperature (*T*_gel_) [29].

#### *In Vitro* Drug Release

Samples of 0.5 ml (500 mcg 5-FU and 250 mcg MTX) were placed in Eppendorf tubes and incubated at 37°C for 1 h for gelation. The formed gels were incubated with 1 ml of phosphate buffer solution (pH 7.4) at 37°C while shaking at 50 rpm. At certain time intervals (0.5, 1, 2, 4, ….., 34 days), 0.5 ml samples were taken and replaced by equal volume of fresh buffer solutions to maintain the sink conditions [30]. The amount of the drug released was estimated by the newly evaluated method for simultaneous analysis of 5-FU and MTX mixture by UV-spectrophotometric method. All measurements were performed in triplicate and the data were reported as means ± SD.

#### Kinetic Treatment of 5-FU and MTX Release Data

The *in vitro* release data were analyzed according various kinetic models including zero order, first order [31], Korsmeyer-Peppas [32], and Higuchi diffusion [33], in order to determine the most appropriate release model that describes the drug release pattern. Model selection was based on the correlation coefficient (*r*) values for the involved parameters as suggested by Burnham and Anderson (Table II) [34].

**Table II:** Kinetic Model Equations

Kinetic models	Equations
Zero order	*Q*_*t*_ = *Q*_0_ − *K*_0_*t*
First order	$\mathit{\log}{Q}_t=\mathit{\log}{Q}_0-\frac{K_1t}{2.303}$
Korsmeyer-Peppas	*logQ*_*t*_ = *logK* + *nlogt*
Higuchi’s equation	${Q}_t={K}_h\sqrt{t}$

*Qt* is the quantity of the drug released at time *t*, *K* is the release rate constant, and *n* is the diffusional exponent that characterizes the best fitted release mechanism)

## RESULTS AND DISCUSSION

### Compatibility Studies

#### Scanning Ultraviolet Absorption Study

The ultraviolet scanning of 1% solution was done using double-beam spectrophotometer, UV-1601 (Shimadzu Co., Japan). It was found that the components used in this study showed no absorbance at the specified selected wave lengths (λ_max_) of 5-FU (266) and MTX (302). On the other hand, the scanning of the 5-FU in the presence of MTX showed certain degree of cross overlapping at the selected absorbance values, which represents a great problem in measuring of 5-FU in the presence of MTX.

#### FTIR Spectroscopy

**Fig. 2:**
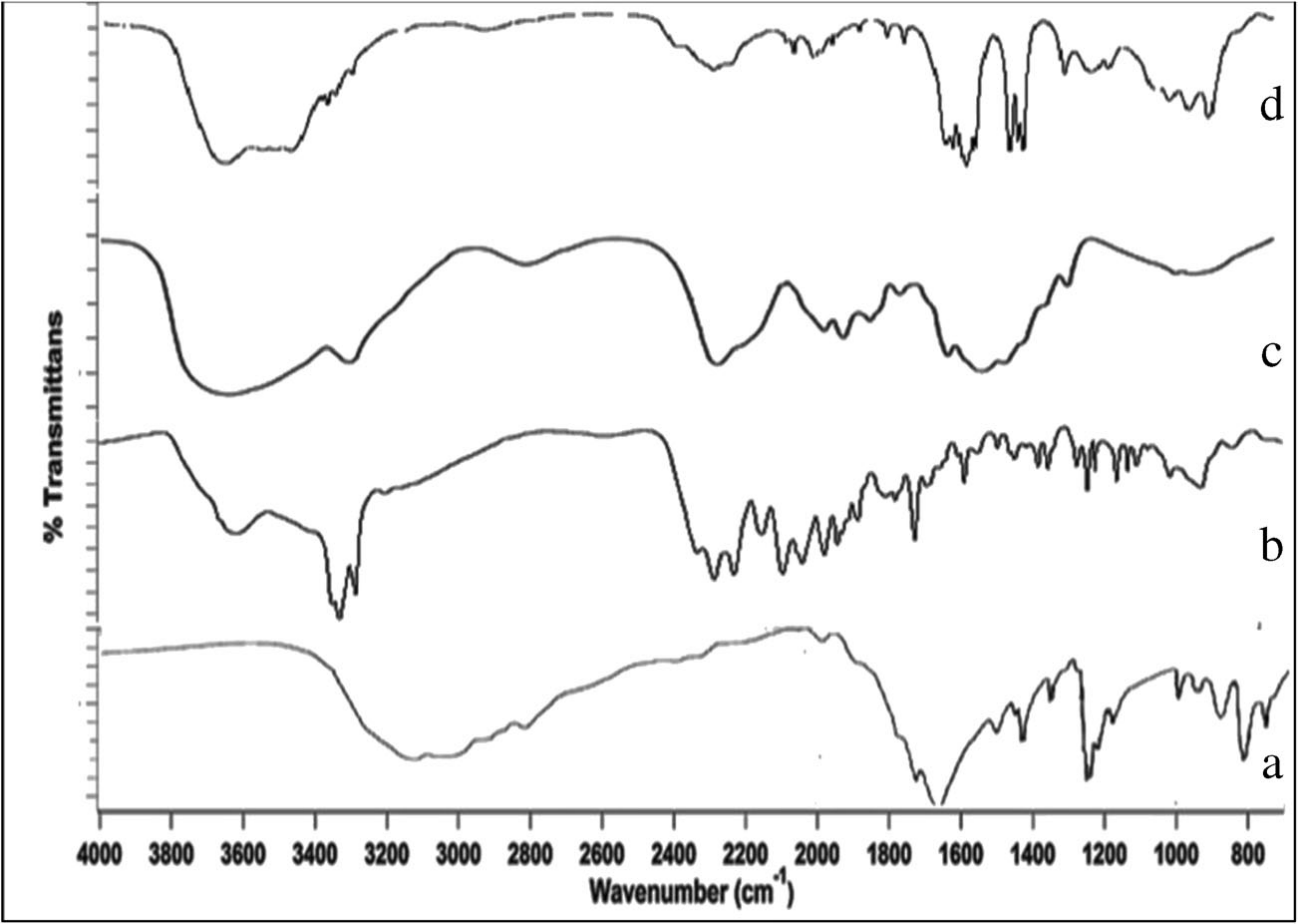
IR absorption spectra of 5-FU (**a**), MTX (**b**), chitosan (**c**) and physical mixture (**d**)

The IR spectra of 5-FU, MTX, chitosan, Pl f127, and physical mixture were recorded using infrared spectrophotometer (IR-476, Shimadzu Co., Japan). Especially, the characteristic bands will be in focus in order to determine the possibility of interaction of 5-FU and MTX with chitosan hydrogel. The IR spectrum of the 5-FU (Fig. 2a) showed characteristic peaks at 1672 cm^−1^ corresponding to C=O stretching vibration, 1431 cm^−1^ due to C–H bending vibration, and 1247 cm^−1^ for C–N stretching vibration. The IR spectrum of MTX (Fig. 2b) showed two characteristic bands at 1603 cm^−1^ and 1624 cm^1^ associated to carboxylate and amide C=O stretching vibration, respectively. Regarding IR spectrum of chitosan (Fig. 2c), it showed characteristic bands at 3420 cm^−1^ for O–H stretch, 2923 cm^−1^ for C–H stretch, 1651 cm^−1^ for C–O stretch of acetyl group, and 1574 cm^−1^ for N–H stretch. These data are in a good accordance with the previously reported data [35–37]. On the other hand, IR spectrum of the physical mixture (Fig. 2d) shows no significant difference in the positions of the absorption bands. The spectra can be simply regarded as combination of both of A, B, and C with the peak position of 5-Fu in the physical mixture having little or no change and slightly diluted. These results indicate that 5-Fu and MTX can be formulated in chitosan hydrogel without interaction with chitosan.

#### Differential Scanning Calorimetry

It has been considered that DSC is a well-developed fast technique for the analysis of drug-drug or drug-excipients interactions. The incompatibilities could be detected by the appearance, shifting, or disappearance of the characteristic endothermic or exothermic peaks and or variations in the corresponding values of enthalpy in the thermograms of drug-excipients mixtures [38].

**Fig. 3:**
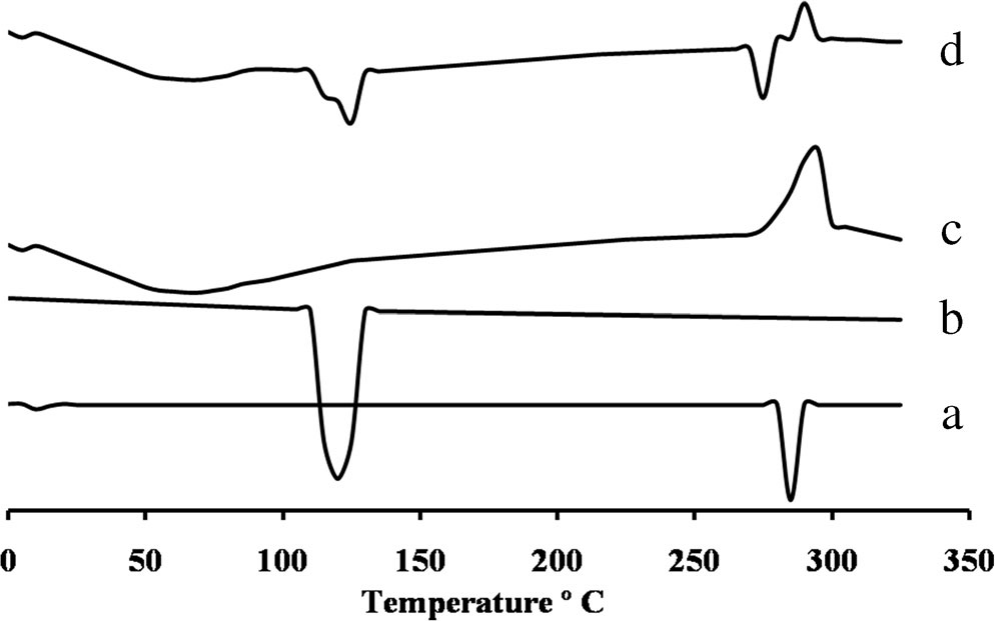
DSC thermal analysis of 5-FU (**a**), MTX (**b**), chitosan (**c**) and physical mixture (**d**)

As shown in Fig. 3a, 5-FU showed a sharp endothermic peak at 285°C corresponding to drug melting, in good agreement with the literature value [39]. DSC thermogram of MTX showed a broad endothermic peak at 122°C corresponds to the melting peak of MTX (Fig. 3b) [40]. DSC curves for purified chitosan (Fig. 3c), showed a broad endothermic peak around 70°C due to the loss of water. An exothermic peak was obtained at 296°C that can be ascribed to the decomposition of amine units [41]. With respect to thermogram of the physical mixture (Fig. 3d), only loss of the peaks intensity with no apparent changes in the characteristic bands of the pure components was observed. The observed short peak around 120°C may be attributed to dilution effect of the sample mixture. Also, forked appearance may be due to in intended contamination of the sample. These result in good accordance with that previously obtained from IR studies.

#### UV-Spectrophotometric Analysis of 5-FU in the Presence of MTX

The results showed that λ_max_ for 5-FU is around 266 nm, where absorbance is weak at 302 nm. On the other hand, MTX has maximum absorption (λ_max_) at 302 nm but its absorption at 266 nm is still of significant value. From the Beer’s Law plots, the slopes (k’s = absorptivity constants) for 5-FU and MTX at the relevant wavelengths were found to be 0.0533, 0.0232, 0.0012, and 0.0259 for K_1f_, K_1m_, K_2f_, and K_2m_, respectively. The concentration of each of the components in the unknown mixture can be calculated by setting up simultaneous equations and solving for the two unknowns as the following [21, 42]:

1 $$ {\mathrm{A}}_1={\mathrm{k}}_{1\mathrm{f}}\ {C}_f+{\mathrm{k}}_{1\mathrm{m}}\ {\mathrm{C}}_{\mathrm{m}}=0.0533\ {C}_f+0.0232\ {\mathrm{C}}_{\mathrm{m}} $$

2 $$ {\mathrm{A}}_2={\mathrm{k}}_{2\mathrm{f}}\ {C}_f+{\mathrm{k}}_{2\mathrm{m}}\ {\mathrm{C}}_{\mathrm{m}}=0.0012\ {C}_f+0.0259\ {\mathrm{C}}_{\mathrm{m}} $$

3 $$ \mathrm{Cm}=\frac{A2-0.0225}{0.0254} $$

4 $$ Cf=\frac{A1-0.0232}{0.0533} $$

*A*_1_ and *A*_2_ are the absorbance of unknown mixture at the first wavelength (266) and the second wavelength (302), respectively. *C*_*f*_ and *C*_*m*_ are the concentration of 5-FU and MTX, respectively.

### Preparation of Thermosensitive Chitosan Hydrogel Systems

**Fig. 4:**
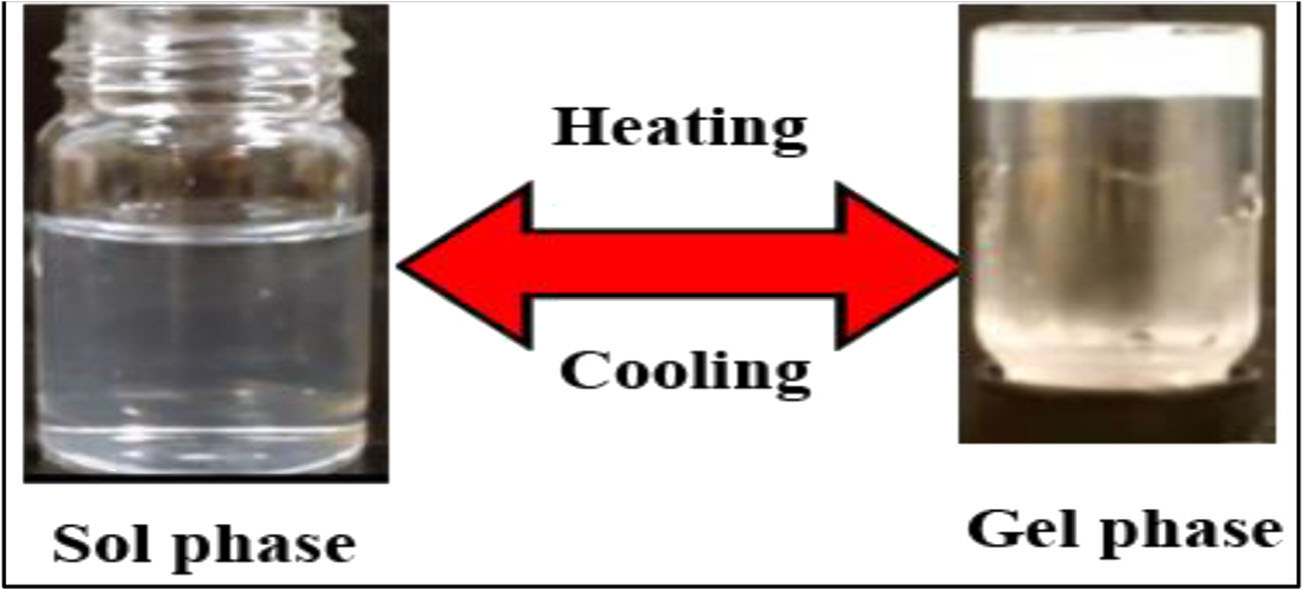
Thermosensitive chitosan hydrogel

All chitosan hydrogel systems were successfully prepared by mixing chitosan solutions with cross-linking agents in an ice path for 15 min (see Fig. 4). The calculated amounts of the drug were dissolved in the prepared solutions by stirring at 500 rpm for 30 min to ensure homogenous distribution of the drug.

### Characterization of the Prepared Chitosan Hydrogels

#### Drug Content Determination

All the prepared hydrogels were tested for their drug content. All investigated formula was found to be within the range of 95–103% (see Table III). These results are considered to be sufficient for further studies.

**Table III:** Physicochemical Properties of Different Chitosan Hydrogel Formulations

Code	Drug content		pH	Viscosity		Gelation point (°C)
	5-FU MTX			Sol (cP) Gel (cP)		
F1	100.5 ± 4.35	102.67 ± 5.22	6.5 ± 1.0	2.117 ± 0.06	114 ± 8	35.5 ± 0.5
F2	103.0 ± 4.94	95.93 ± 2.41	6.36 ± 0.06	2.123 ± 0.03	117.8 ± 1.04	24.3 ± 1.1
F3	102.17 ± 5.93	102.53 ± 2.0	7.3 ± 0.06	3.292 ± 0.07	153 ± 4.58	19.0 ± 1.0
F4	102.0 ± 3.80	98.08 ± 5.16	6.7 ± 0.17	2.449 ± 0.04	120.8 ± 1.26	29.3 ± 1.5

#### Visual Inspection (Organoleptic Properties)

The prepared chitosan hydrogels were visually examined for their organoleptic properties before and after gelation. The results showed that all the systems were clear and uniform with no lumps or phase separation in both sol and gel forms. The color was white and turbid in the gel form and clear colorless in the sol form. These results indicate the validity of the prepared hydrogels for parental administration.

#### pH Determination

It was observed that the pH of the prepared formulae F1, F2, and F4 was in the range of (6.2 to 6.9) and that of F3 was 7.3 (see Table III) indicating the utility of all formulations for subcutaneous injection since. The higher pH value of F3 may be attributed to alkali imparting component (Na_2_CO_3_) of hydroxyapatite (Ca_5_(PO_4_)_3_(OH) cross-linking agent. It was reported that pH of injectable hydrogels should lie in a pH range of 6.5–7.4 to avoid cell damage [43].

#### Viscosity Measurement

The viscosity of the prepared hydrogels in both sol and gel states was measured and is presented in Table III. The results showed that with the exception of F3 solution, all hydrogel solutions having certain degree of similarity in their rheological behaviors. This is because water is the major component in all formulations. However, in the case of F3 solution, higher viscosity may be attributed to higher viscosity of hydroxyapatite solution at low temperature as well as stronger interaction with chitosan solution after mixing [44]. Similarly for gel forms, the viscosity values were higher from F3 hydrogels compared to those of F1, F2, and F4 hydrogels. Noteworthy, sol-gel transition phenomenon is the major requirement for injectable hydrogel to provide certain degree of syringeability and injectability [45].

#### Syringeability and Injectability

The prepared chitosan solutions cross-linked with β-GP (F1), 20% Pl F127 (F2), or β-GP-10% Pl F127 (F4) were continuously extruding 0.5 cm within 5–10 s when 5 mm syringe was pressed with fingers with mild to moderate force. These results indicate smooth and easy injection of these solutions from the syringes to the meat sample. On the other hand, chitosan/HA solution (F3) shows high degree of resistance to flow from the needle. This may be attributed to the high viscosity of this formula compared to β-GP and PL F127 chitosan formulations [46].

#### Sol-Gel Transition Temperature Measurement

All the prepared chitosan solutions undergo the sol to gel transition by changing the temperature (Table III). The mechanisms of these sol-to-gel transitions were believed to be shifting in equilibrium from unimer to micelle, micellar growth, or by micellar expansion accompanying an increase in aggregation number driven by hydrophobic or attractive forces [29, 44].

#### *In Vitro* Drug Release

**Fig. 5:**
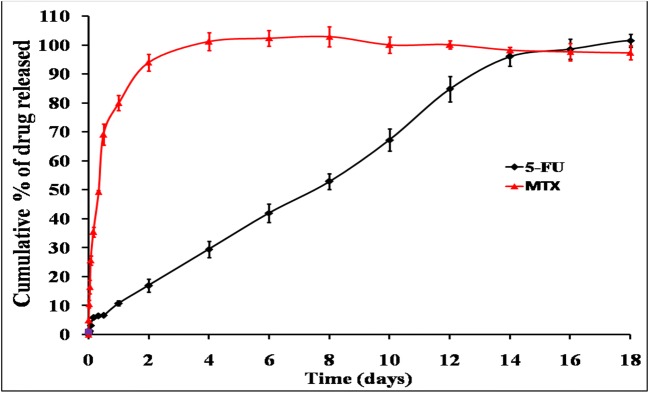
*In vitro* release profile of 5-FU an MTX from chitosan/β-GP hydrogel system (F1)

**Fig. 6:**
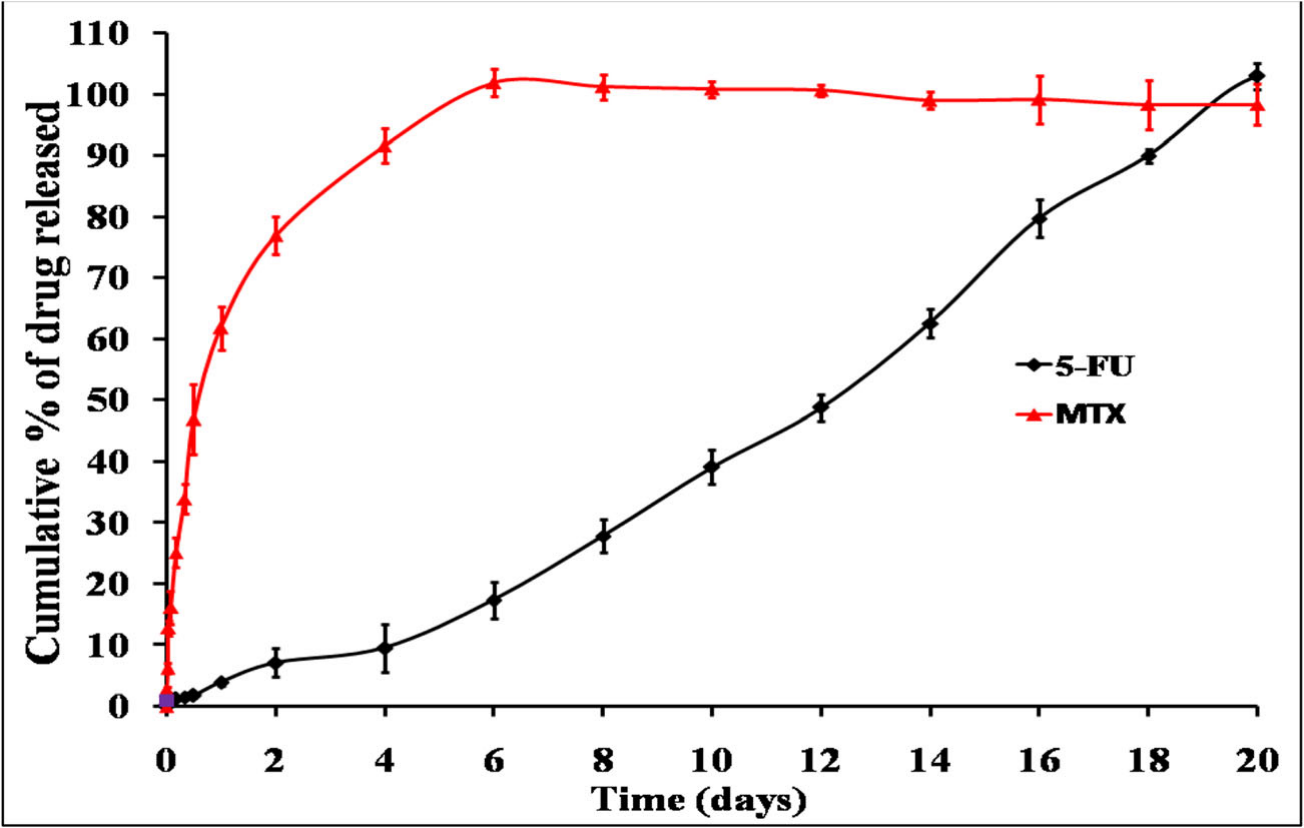
*In vitro* release profile of 5-FU an MTX from chitosan/Pluronic F127 hydrogel system (F2)

**Fig. 7:**
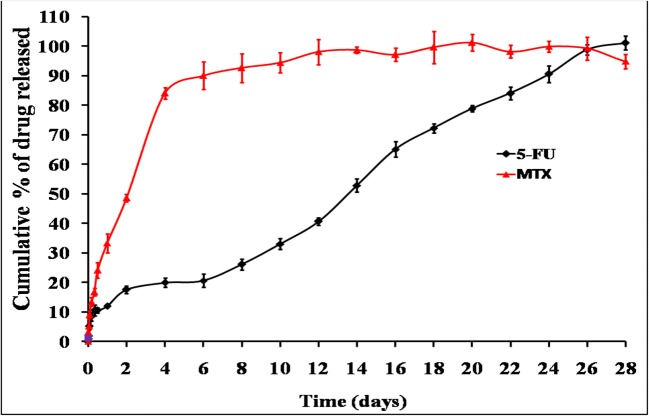
*In vitro* release profile of 5-FU an MTX from chitosan/HA hydrogel system (F3)

**Fig. 8:**
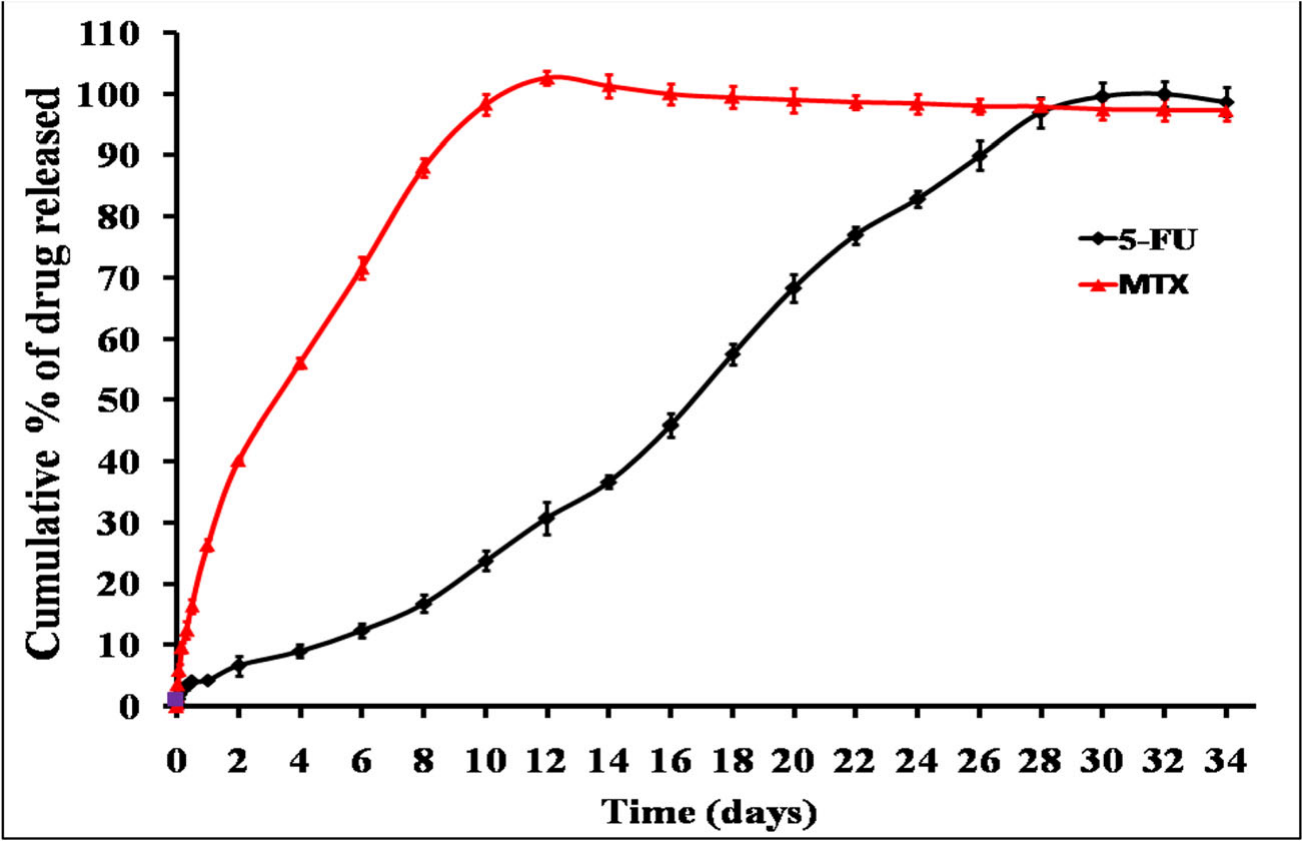
*In vitro* release profile of 5-FU an MTX from chitosan/β-GP/Pluronic F127 hydrogel system (F4)

In order to understand the ability of different chitosan systems to effectively deliver 5-FU and MTX in a sustained fashion, *in vitro* release studies were performed in phosphate buffer solution (pH 7.4) at 37°C under sink conditions. These conditions ensure that there are no changes in the previously measured physicochemical properties including viscosity and gelation point. The release profiles of 5-FU and MTX from different chitosan hydrogels are graphically illustrated in Figs. 5, 6, 7, and 8. Generally, the obtained results indicated that the release rate of MTX from different chitosan systems was higher than that of 5-FU (more than 50% of drug released within the first 2 days in all formulations). This order of drug release rates is intended to provide the synergistic effect of 5-FU and MTX combination, and it may be attributed to higher solubility of MTX with respect to 5-FU [4]. Regarding the effect of different chitosan hydrogel systems on the release profile of 5-FU, the results showed that the percentage of drug release was the highest from F1 hydrogel (95.3% within 2 weeks). This may be due to the lower viscosity of chitosan-β-GP hydrogel system compared to other systems (see results of rheological characterization). The release of 5-FU from other chitosan hydrogel systems was arranged in the following descending order: F1 ***>*** F2 ***>*** F3 ***>*** F4.

On the other hand, the longest release of 5-FU was obtained from chitosan hydrogel system containing β-GP and 10% PL F127 combination (F4) which provided sustained release for more than 1 month in a good similarity with the chemotherapeutic cycle [47]. This finding can be explained by the higher affinity of the drug molecules to the hydrogel base if it is added during the formulation step which, consequently, hinders its release [48]. All experiments were carried out until complete drug release to exclude the possibility of drug engagement by the hydrogel base.

#### Kinetic Treatment of 5-FU and MTX Release Data

**Fig. 9:**
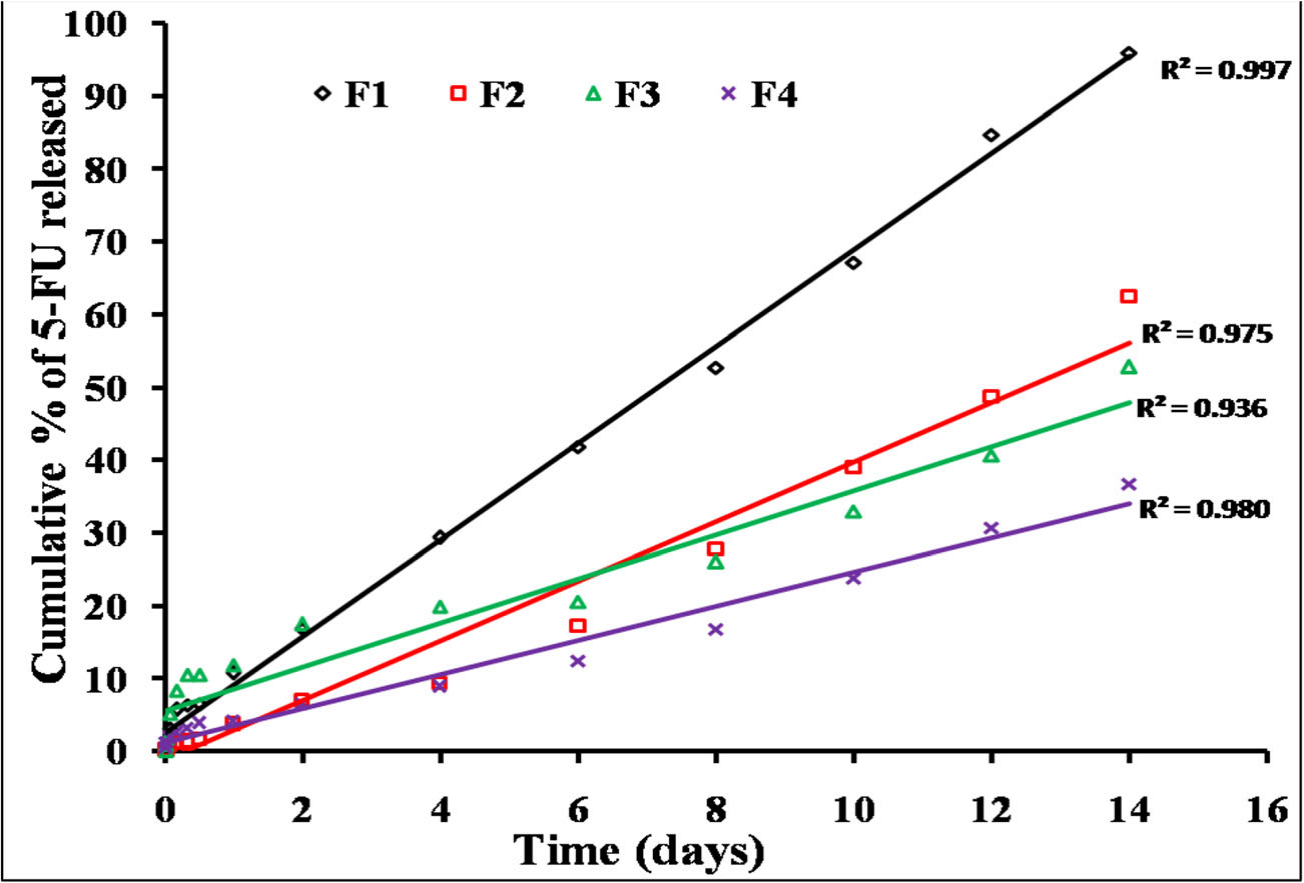
Kinetics release profiles of 5-FU from different chitosan hydrogels (F1, F2, F3, and F4) plotted according to zero-order mechanism

**Fig. 10:**
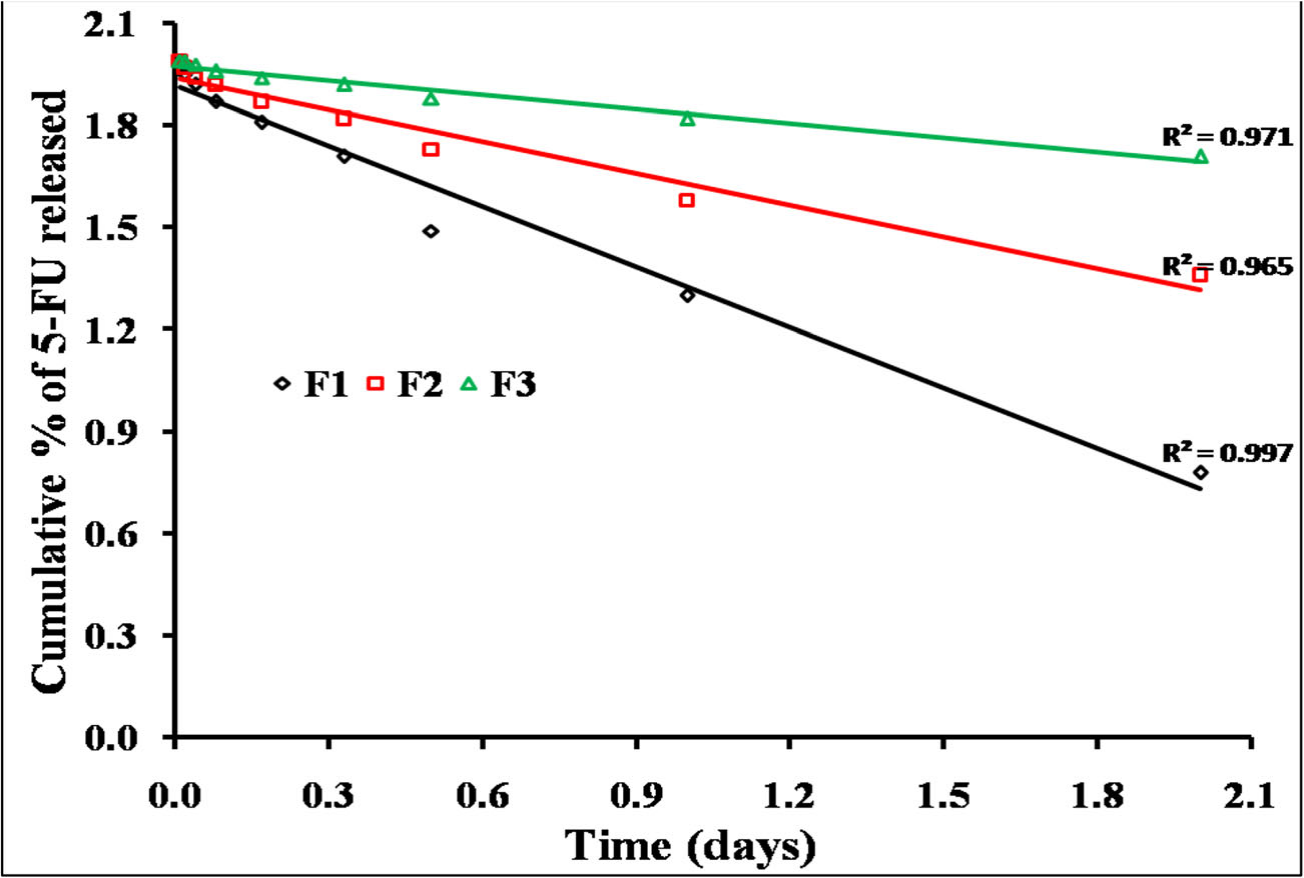
Kinetics release profiles of MTX from different chitosan hydrogels (F1, F2, and F3) plotted according to first-order mechanism

**Fig. 11:**
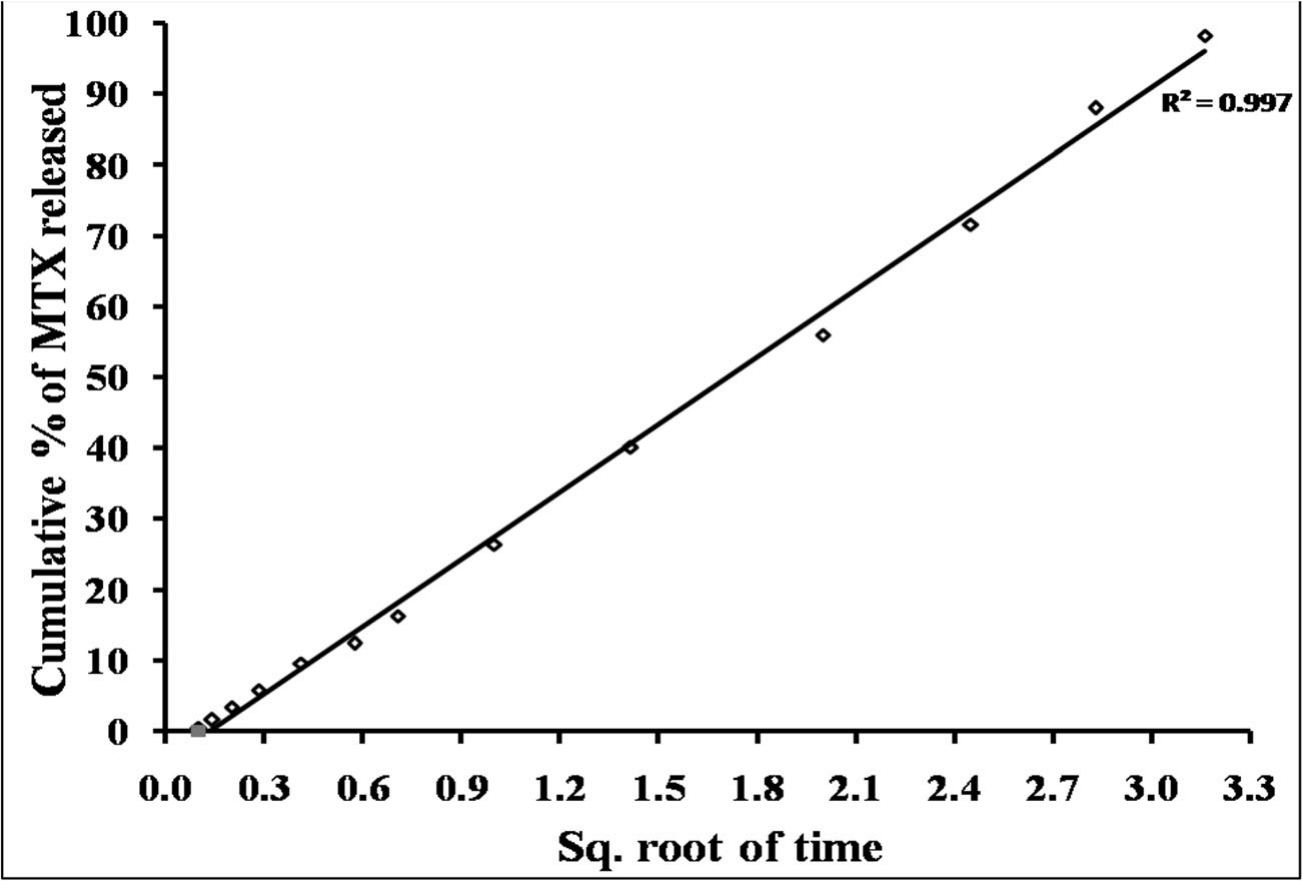
Kinetics release profile of MTX from chitosan hydrogel (F4) plotted according to Higuchi diffusion mechanism

The release kinetic order of 5-FU and MTX from differently prepared chitosan hydrogel was determined simply using graphical representation methods [31]. The results indicate that the release of MTX from different chitosan hydrogels follows first-order kinetics in some formulations (F1, F2, and F3) and Higuchi diffusion model in F4. These results may be attributed to higher solubility of MTX which is used in its lyophilized forms. Thus, the released amounts directly affected by the initial amount in the formulae. On the other hand, the release of 5-FU from different hydrogel formulations follows zero-order kinetics. These results suggested that chitosan hydrogel plays an important role in controlling release of the drug to the surrounding tissues (Figs. 9, 10, and 11; Table IV) [31].

**Table IV:** Kinetic Data for Percentage of 5-FU and MTX Released from Different Hydrogel Systems

Kinetic models		Different hydrogel systems							
		F1		F2		F3		F4	
		5-FU	MTX	5-FU	MTX	5-FU	MTX	5-FU	MTX
Zero order	r	*0.997*	0.783	*0.973*	0.798	*0.983*	0.844	*0.973*	0.962
	Slope	6.642	4.30	4.683	2.156	3.586	8.628	3.302	9.857
First order	r	0.855	0.978	0.838	*0.977*	0.762	*0.987*	0.858	0.904
	Slope	0.074	0.593	0.041	0.261	0.045	0.137	0.029	0.141
Korsmeyer-Peppas	r	0.987	0.964	0.964	0.952	0.906	0.979	0.940	0.970
	Slope	0.128	0.474	0.131	0.267	0.062	0.560	0.607	0.689
Higuchi diffusion	r	0.948	0.947	0.861	0.960	0.914	0.962	0.858	*0.997*
	Slope	2.400	7.093	1.849	4.868	1.740	3.170	1.561	3.170
Best fitted model		Zero	First	Zero	First	Zero	First	Zero	Higuchi

## Conclusion

Different chitosan hydrogel systems were successfully prepared. Chitosan hydrogel prepared using combination of β-Gp and 10% Pl F127 as cross-linking agents (F4) was found to have the longest duration of action (over 4 weeks) with good physicochemical properties, including pH, viscosity, syringeability, and injectability. Since the monthly injection of drug will be more convenient than frequent application; this system can be considered as a promising vehicle for quantitative release of anti-cancer drugs in a sustained manner.
